# Anti-*Salmonella* Activity of *Thymus serpyllum* Essential Oil in Sous Vide Cook–Chill Rabbit Meat

**DOI:** 10.3390/foods13020200

**Published:** 2024-01-08

**Authors:** Miroslava Kačániová, Natália Čmiková, Maciej Ireneusz Kluz, Boutheina Ben Akacha, Rania Ben Saad, Wissem Mnif, Bożena Waszkiewicz-Robak, Stefania Garzoli, Anis Ben Hsouna

**Affiliations:** 1 Institute of Horticulture, Faculty of Horticulture and Landscape Engineering, Slovak University of Agriculture, 94976 Nitra, Slovakia; n.cmikova@gmail.com; 2 School of Medical & Health Sciences, University of Economics and Human Sciences in Warsaw, 01 043 Warszawa, Poland; m.kluz@vizja.pl (M.I.K.); b.waszkiewicz-robak@vizja.pl (B.W.-R.); 3 Laboratory of Biotechnology and Plant Improvement, Centre of Biotechnology of Sfax, Sfax 3018, Tunisiaraniabensaad@gmail.com (R.B.S.); benhsounanis@gmail.com (A.B.H.); 4 Department of Chemistry, College of Sciences at Bisha, University of Bisha, Bisha 61922, Saudi Arabia; wmoneef@ub.edu.sa; 5 Department of Chemistry and Technologies of Drug, Sapienza University, 00185 Rome, Italy; stefania.garzoli@uniroma1.it; 6 Department of Environmental Sciences and Nutrition, Higher Institute of Applied Sciences and Technology of Mahdia, University of Monastir, Monastir 5000, Tunisia

**Keywords:** *Longissimus dorsi muscle*, rabbits, wild thyme essential oil, *Salmonella enterica* subsp. *enterica*, sous vide, microbiological quality

## Abstract

Food is generally prepared and vacuum-sealed in a water bath, then heated to a precise temperature and circulated in a sous vide machine. Due to its affordability and ease of use, this cooking method is becoming increasingly popular in homes and food service businesses. However, suggestions from manufacturers and chefs for long-term, low-temperature sous vide cooking raise questions about food safety in the media. In this study, heat treatment with different times and wild thyme essential oil (EO) in sous vide-processed rabbit *longissimus dorsi muscle* were found to inactivate *Salmonella enterica*. The rabbit meat samples were vacuum-packed in control groups, in the second group the rabbit meat samples were injected with *S. enterica*, and in the third group were meat samples infected with *S. enterica* with *Thymus serpylum* EO additive. The vacuum-packed samples were cooked sous vide for the prescribed time at 55, 60, and 65 °C. At 5, 15, 30, and 60 min, the quantities of *S. enterica*, total bacterial counts, and coliform bacteria were measured in groups of sous vide rabbit meat. Microbiological analyses of rabbit meat samples on days 1 and 7 were evaluated. In this study, total viable counts, coliforms bacteria, and number of *Salmonella* spp. were identified. After incubation, isolates from different groups of microorganisms were identified by the mass spectrometry technique. For each day measured, the test group exposed to a temperature of 55 °C for 5 min had a greater number of total microbiota. The most isolated microorganisms by MALDI-TOF MS Biotyper from the control and treated groups were *Lactococcus garvieae* and in the treated groups also *S. enterica*. Based on our analysis of sous vide rabbit meat samples, we discovered that adding 1% of thyme essential oil to the mixture reduced the amount of *Salmonella* cells and increased the overall and coliform bacterial counts. The microbiological quality of sous vide rabbit meat that was kept for seven days was positively impacted by the addition of thyme essential oil.

## 1. Introduction

In general, rabbit meat is a widely consumed food. In recent years there has been a growth in its use in several countries in Europe, the Middle East, and in some North African countries, particularly in Egypt [1]. Due to a variety of factors, it constitutes a good healthy dietary source for human nutrition. Its meat is characterized by low levels of fat, cholesterol, and sodium content, as well as high levels of biologically valuable proteins and polyunsaturated fatty acids (PUFA). Additionally, it contains a range of crucial macro- and micronutrients, such as the minerals potassium, phosphorus, and selenium, as well as vitamins such as B_12_ [2,3].

Worldwide, *Salmonella* continues to be a leading cause of foodborne illness among people. Additionally, it can infect commercial rabbits; pregnant does and young bunnies are particularly vulnerable. *Salmonella Typhimurium* or *Enteritidis* are linked to the majority of rabbit salmonellosis outbreaks in Spain [4]. Based on fecal samples, a small number of surveys indicate that less than 1% of healthy rabbits at the slaughterhouse test positive for *Salmonella* [5,6]. Acute enteritis in rabbits caused by salmonellosis is a rare disease characterized by rapid mortality and infertility, with abortions often occurring in pregnant rabbits [7]. The EU’s successful *Salmonella* control program in the food chain is responsible for the general trend in the decline in the prevalence of *Salmonella* in meats [8,9]. Public health is still at risk, meanwhile, because raw meats, raw minced meats, and prepared foods with *Salmonella* can still be found in retail stores. The pathogen’s transmission from the animal during slaughter is responsible for the frequency of *Salmonella* in meat, underscoring the need to follow sanitary procedures in the meat production process [10].

A technique called sous vide (SV) involves cooking raw materials previously vacuum-packaged with strictly regulated time and temperature restrictions. Its main application until 2011 was to increase the shelf life of food goods. In this case, its application involves the combination of techniques that incorporate a series of parallel fixing elements, including vacuum conditions, low temperature, and extended process time. Due to the cumulative nature of these parameters, they are more effective in confined negative changes than in prolonged process periods separately [11]. It is currently one of the culinary processing techniques used in catering facilities to develop new meals [12]. Unlike conventional procedures, the sous vide method uses temperatures that typically range from 50 to 85 °C and requires a longer cooking period (between 2 and 48 h). The type of meat, size of the cut, intramuscular connective tissue, and myofibrillar protein components all affect how it is heated [13].

A combination of five *Salmonella* strains was tested for heat resistance in chicken breasts marinated in teriyaki. The inoculated meat, wrapped in bags, was submerged whole in a water bath that was continuously moved. It took one hour to reach a final temperature of 55, 57.5, or 60 °C, after which it was kept for predetermined periods of time. *Salmonella* D values in chicken breast ranged from 47.65 min at 55 °C to 7.48 min at 60 °C, as determined by linear regression. The pathogen became more susceptible to the lethal effects of heat after marinating. The findings of this study will help the food sector to guarantee the microbiological safety of marinated chicken breasts that are processed by the sous vide method [14]. Sage EO and *S. enterica* were combined to see if heat treatment would improve the effectiveness of sous vide. The samples were vacuum packed, injected with *S. enterica*, and then cooked by the sous vide method for the required time at 50–65 °C. After a 20-min heat treatment at 65 °C, the samples were generally free of bacteria and coliforms. The stabilization and safety of beef tenderloin were shown to be achieved by combining the sous vide method with the addition of sage essential oil (EO).

The *Thymus* EOs (TEOs) have been discovered to be effective against microbiological pathogens, particularly those that cause infections in plants, baked goods, meat and dairy products, and food commodities that are kept in storage [15]. Since TEOs are safer substitutes for synthetic preservatives, food preservatives based on EOs have mostly protected food commodities from biodeterioration and spoiling in the current food system [16]. Because the scents of the EO do not negatively impact the organoleptic qualities of these kinds of meals, TEO has been utilized to preserve meat products against microbial contaminations [17]. Interestingly, bacterial resistance or adaptation has not been documented in EO-based antibacterial compounds, and their side effects are minor, making them very important. The death rate of *S. enterica* on raw chicken meat is markedly increased by the addition of thyme oil at 0.5 or 1.0% (*v*/*v*) [18]. As a result, in comparison to the control, TEO decreased the final *Salmonella* counts by 0.52–2.82 log CFU/g. When TEO was used at lower concentrations (0.3% and 0.6%), *Salmonella* counts were reduced by more than 2 log CFU/g; at higher doses (0.9%), the reduction was as much as 4 log CFU/g. The combination of EOs and meat components can account for the greater anti-*Salmonella* effects of higher TEO concentrations compared to lower values [19].

In recent years, more sous vide foods have been prepared at low temperatures, considered a “temperature danger zone” in the industry. Safety assessment is made more difficult by the fact that its impact on bacterial behavior has not been thoroughly examined [20]. Low cooking temperatures and inadequate sous vide application can prevent the inactivation of harmful organisms and promote their growth. *Listeria monocytogenes* [14,21], *Clostridium perfringens* spores [20], and *Salmonella* spp. [22] in low-temperature long-time processing treated chicken breast and other animal products [23] have been predicted to survive based on specific prognostic models.

Therefore, the goal of this study was to determine the effect of commercially available *Thymus serpyllum* essential oil on the thermal inactivation of *Salmonella enterica* in sous vide rabbit *longissimus dorsi muscle* samples at 55, 60, and 65 °C.

## 2. Materials and Methods

### 2.1. Preparation of Bacterial Suspension

The test was conducted using *Salmonella enterica* subsp. *enterica* CCM 4420 (Czech Collection of Microorganisms, Brno, Czech Republic). The bacterial inoculum was grown for 24 h at 37 °C on Mueller Hinton agar (MHA, Oxoid, Basingstoke, UK). One hundred microliters of inoculum was introduced to the rabbit meat samples after the inoculum’s optical density was adjusted to the 0.5 McFarland standard (1.5 × 10^8^ CFU/mL). To guarantee that the pathogen was distributed properly, samples of rabbit flesh were injected with *S. enterica* and homogeneously mixed for three minutes at room temperature.

### 2.2. Essential Oil

Hanus s.r.o. in Nitra, Slovakia provided the *Thymus serpyllum* EO, produced by steam distillation of dried flowering stem. It was stored at 4 °C in the dark before the analysis. Gas chromatography/mass spectrometry (GC/MS) and gas chromatography/flame detector ionization (GC-FID) techniques were used to characterize the chemical profile of *T. serpyllum* EO. The major components were thymol (18.8%), carvacrol (17.4%), o-cymene (15.4%), and geraniol (10.7%) [24].

### 2.3. Sample of Rabbit Meat Preparation

In this investigation, samples of rabbit meat from the *longissimus dorsi muscle* were used. The Slovak Republic’s label information states that the meat sample was taken from a New Zealand breed that was bought from a licensed store in Slovakia (Nitra, 48°18′27.47′′ N 18°05′4.31′′ E). The rabbit meat’s physical and chemical characteristics were as follows: protein content 20%, fat content 3.4%, and pH 6.26. One thousand two hundred grams of rabbit flesh in all were collected, and they were refrigerated before being delivered to the microbiological lab. The rabbit meat was then divided into 5 g chunks, each of which was weighed after being cut using a sterile knife. A total of 219 samples of 5 g weight were prepared. Three raw rabbit meat samples, 36 control and treated samples of rabbit meat on day 1, and 36 control and treated samples of rabbit meat on day 7 were used. Using a vacuum packer (Concept, Choce, Czech Republic), samples of 5 g of chopped meat were the control and the treated groups, which also contained 1% (*v*/*w*) *T. serpyllum* EO solution, dissolved in rapeseed oil, and were then vacuum wrapped. Each sample of rabbit meat (5 g) was packaged individually. The groups of control samples were vacuum-packed. The samples with the addition of EO and *Salmonella* were prepared, and rabbit meat samples were put into the main bags and mixed gently in the bag for 1 min approximately, so as not to damage the meat sample, with the addition of 100 µL *Salmonella* and EO 1% (*v*/*w*). After this operation, they were vacuum-packed.

In our trial, the following was made available:

RM: For anaerobic storage, fresh rabbit meat was vacuum sealed in polyethylene vacuum cooking bags and kept at 4 °C before being heated to 55–65 °C for 5–60 min.

RMSE: Fresh rabbit meat was vacuum sealed in polyethylene vacuum cooking bags, mixed with *S. enterica*, and kept anaerobically at 4 °C before being heated to 55–65 °C for five to sixty minutes.

RMSEEO: Fresh rabbit meat was vacuum-packed into polyethylene vacuum cooking bags, treated with 1% wild thyme EO, mixed with *S. enterica*, and kept at 4 °C before heating to 55–65 °C for 5 to 60 min.

Raw, uncooked rabbit meat was used to prepare the control samples on day zero. The essential oil in the first group of samples and *S. enterica* in the second group of samples were applied to the samples, mixed gently with samples, and maceration was performed on them for 24 h. The samples were cooked on the CASO SV1000 sous vide apparatus (Arnsberg, Germany). Individual samples were divided into groups, while they were heat-treated under controlled conditions of temperature and time for sous vide preparation. Polyethylene high barrier bags for vacuum packaging from material at 40 to 200 microns that are impermeable, protect against moisture, are extremely temperature resistant: −30 °C to +100 °C, show no softening of weld seams, have particularly long durability guaranteed, are suitable for refrigeration and freezer storage for several years, food safe, tasteless, and odorless, 100% free of plasticizers (e.g., bisphenol A) according to the data sheet, 100% BPA free, and are 100% free of microplastics were used.

### 2.4. Cultivation of Samples

On days 1 and 7, microbiological analyses were performed. Before heating, samples were stored at a temperature of 4 °C for 24 h, and after heating, half of the samples were evaluated after 7 days. A quantity of 5 g of meat samples was weighed and thereafter placed in an aseptic stomacher bag. After that, 45 mL of peptone water was used to dilute the samples to 10^−1^, and a stomacher was used to homogenize them for two minutes. Then, 0.1 mL of an aliquot from an appropriate dilution was pipetted and spread on standard pre-dried plate count agar media. These microbial species were evaluated: to grow coliform bacteria (CB) the Violet Red Bile Lactose Agar (VRBL; Oxoid, Basingstoke, UK) incubated at 37 °C for 24 for 48 h, was used. To grow total viable counts (TVC), Plate Count Agar (PCA; Oxoid, Basingstoke, UK), incubated at 30 °C for 48–72 h, was used. The 24 to 48 h incubation period was carried out on Xylose Lysine Deoxycholate Agar (XLD; Oxoid, Basingstoke, UK) for the *Salmonella* spp. growth. After incubation and enumeration from each plate, 8 different colonies on Tryptone Soya agar (TSA; Oxoid, Basingstoke, UK) were reinoculated and incubated for 30 °C. The methods were previously described by Gál et al. [25].

### 2.5. Microorganisms Identification with Mass Spectrometry

Microorganisms isolated from samples of rabbit flesh were identified using the MALDI-TOF (Matrix-Assisted Laser Desorption/Ionization Time of Flight) MS Biotyper (Bruker, Daltonics, Bremen, Germany) and reference libraries.

A stock solution was produced, and it became an organic material. The standard solution consisted of 2.5% trifluoroacetic acid, 47.5% water, and 50% acetonitrile. Five hundred microliters of pure 100% acetonitrile, 475 µL of filtered water, and 25 µL of pure 10% trifluoroacetic acid were combined to create 1 mL of stock solution. The “HCCA matrix portioned” was produced and combined with the organic solvent in a 250 µL Eppendorf flask. All matrix materials were purchased from Aloqence Science (Vrable, Slovakia).

Previous recommendations were followed when creating the samples [26]. The Petri dish included eight distinct colonies, which were chosen. Nine hundred milliliters of ethanol was added after the biological substance was transferred from a Petri plate to an Eppendorf flask along with 300 mL of distilled water, stirred, and then the mixture was centrifuged for two minutes at 10,000× *g* using an ROTOFIX 32A (Ites, Vranov, Slovakia). The precipitate was removed from the Eppendorf tube and left to dry at room temperature (20 °C) after the supernatant was discarded. The particle was then exposed to 30 mL of 70% formic acid and 30 mL of acetonitrile. The mixture was then centrifuged for two minutes at 14,000 rpm. A MALDI plate was coated with 1 mL of liquid, and then 1 mL of MALDI matrix solution was added. The samples were dried before being examined for the presence of microorganisms in a MALDI-TOF mass spectrometer (Bruker, Daltonics, Bremen, Germany). The Microflex LT MALDI-TOF mass spectrometer (Bruker Daltonics, Bremen, Germany) was set to operate in the linear positive mode with a mass range of 2.000–20.000 Da, and it was used to automatically generate mass spectra. The Bruker bacterial test standard was used to calibrate the equipment. With the use of the MALDI Biotyper 3.0 program (Bruker Daltonics, Bremen, Germany), the mass spectra results were evaluated. Identification criteria were as follows: scores between 2.300 and 3.000 indicated extremely probable species identification, between 2.000 and 2.299 indicated genus identification with plausible species identification, between 1.700 and 1.999 indicated probable genus identification, and a score of less than 1.700 was considered an unreliable identification.

### 2.6. Statistical Analyses

All measurements were conducted following 1 and 7 days of storage, and each analysis and test were performed in triplicate. Prism 9 software (GraphPad Software, San Diego, CA, USA) was utilized to compute the mean values and standard deviations (SD) for microbial counts. A two-way analysis of variance (ANOVA) was performed at a significance level of 0.05 using Prism 9. Subsequently, Tukey’s test was applied for figures involving three variables (RM vs. RMSE, RM vs. RMSEEO, RMSE vs. RMSEEO), while Šídák’s multiple comparisons test was employed for figures involving two variables (RMSE and RMSEEO).

## 3. Results

### 3.1. Microbiological Analyses of Rabbit Sous Vide Meat at 1 and 7 Days

**Figure 1:**
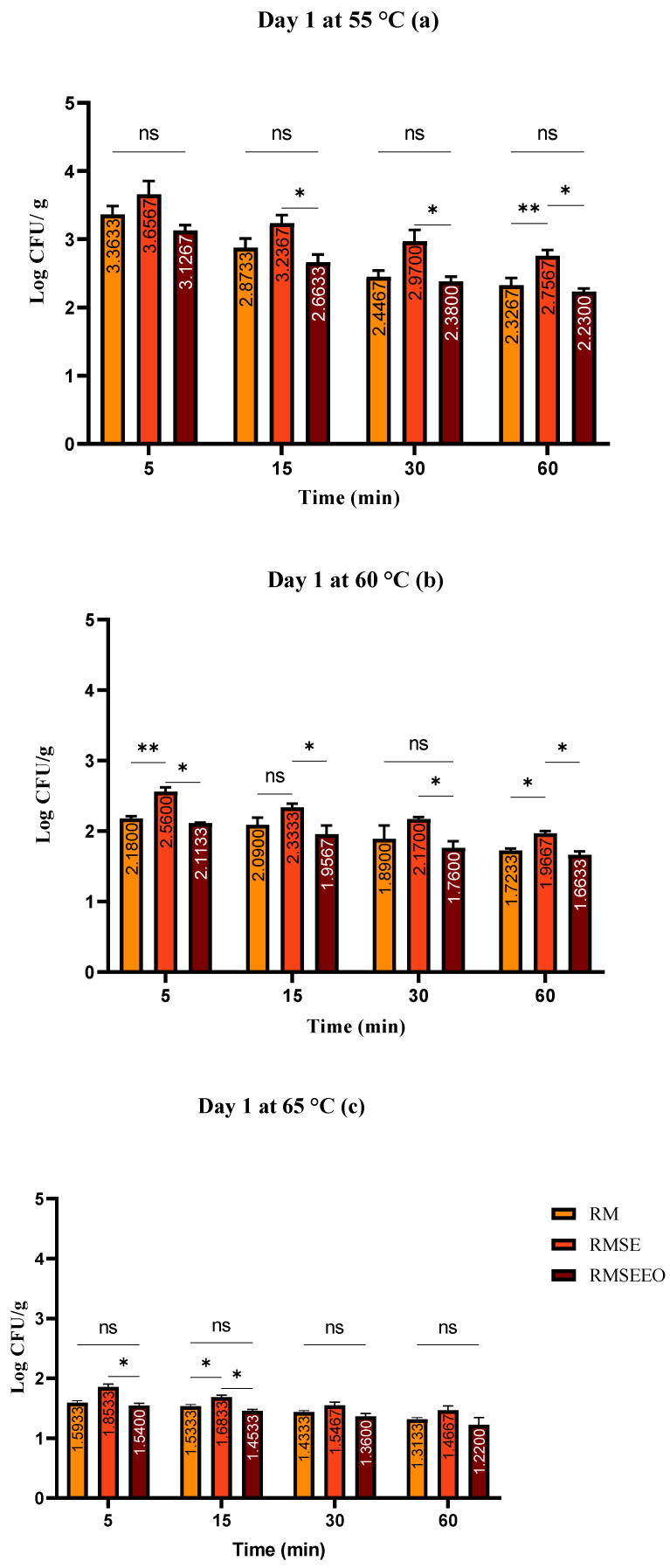
(**a**–**c**). Total count of bacteria (log CFU/g) at day 1: Tukey’s multiple comparisons test Groups (RM vs. RMSE, RM vs. RMSEEO, and RMSE vs. RMSEEO), ** *p* ≤ 0.01, * *p* ≤ 0.05, ns—not significant.

Raw, uncooked rabbit meat was used to prepare the control samples and evaluated on day 0, where a total count of bacteria with a value of 3.17 ± 0.01 log CFU/g and coliform bacteria with a value of zero were found. The effects of wild thyme EO for each heat treatment on day one sous vide rabbit samples are shown in Figure 1a–c. This figure shows the average counts found in samples with or without wild thyme EO over time and consistent with thermal treatment. Overall bacterial counts were higher in samples heated to 55 °C for five minutes. The total number of bacteria in the control groups ranged from 1.31 ± 0.04 to 3.36 ± 0.12 log CFU/g. The highest total bacteria counts were found in *S. enterica*-treated groups.

**Figure 2:**
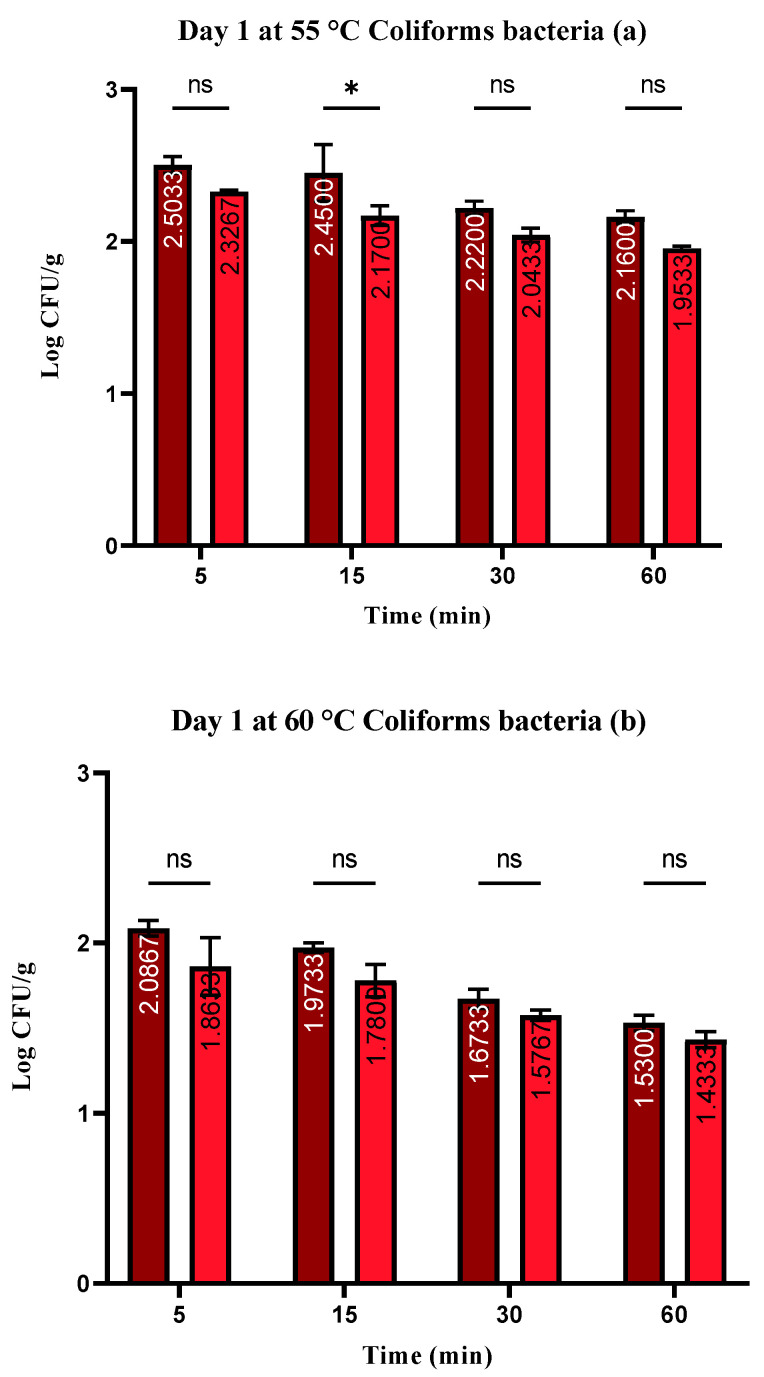

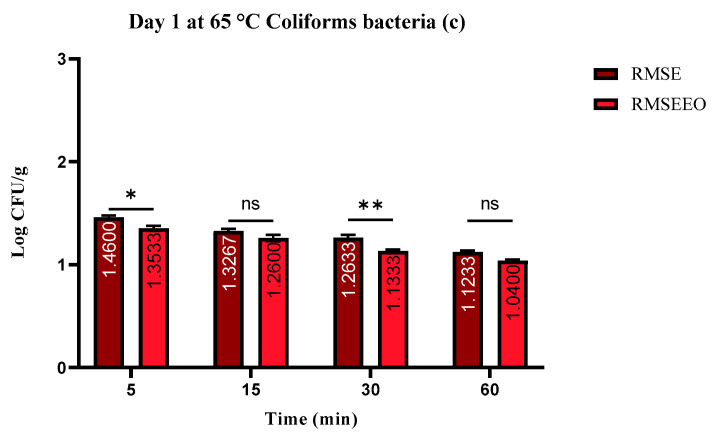
(**a**–**c**). Number of coliform bacteria (log CFU/g) at day 1: Šídák’s multiple comparisons test; Group (RMSE—RMSEEO), ** *p* ≤ 0.01, * *p* ≤ 0.05, ns—not significant.

No coliform bacteria were found in the control group and in the group treated with *S. enterica* they ranged from 1.12 ± 0.03 to 2.45 ± 0.05 log CFU/g and in the group treated with wild thyme EO and *S. enterica* they ranged from 1.14 ± 0.03 to 2.17 ± 0.05 log CFU/g (Figure 2a–c).

**Figure 3:**
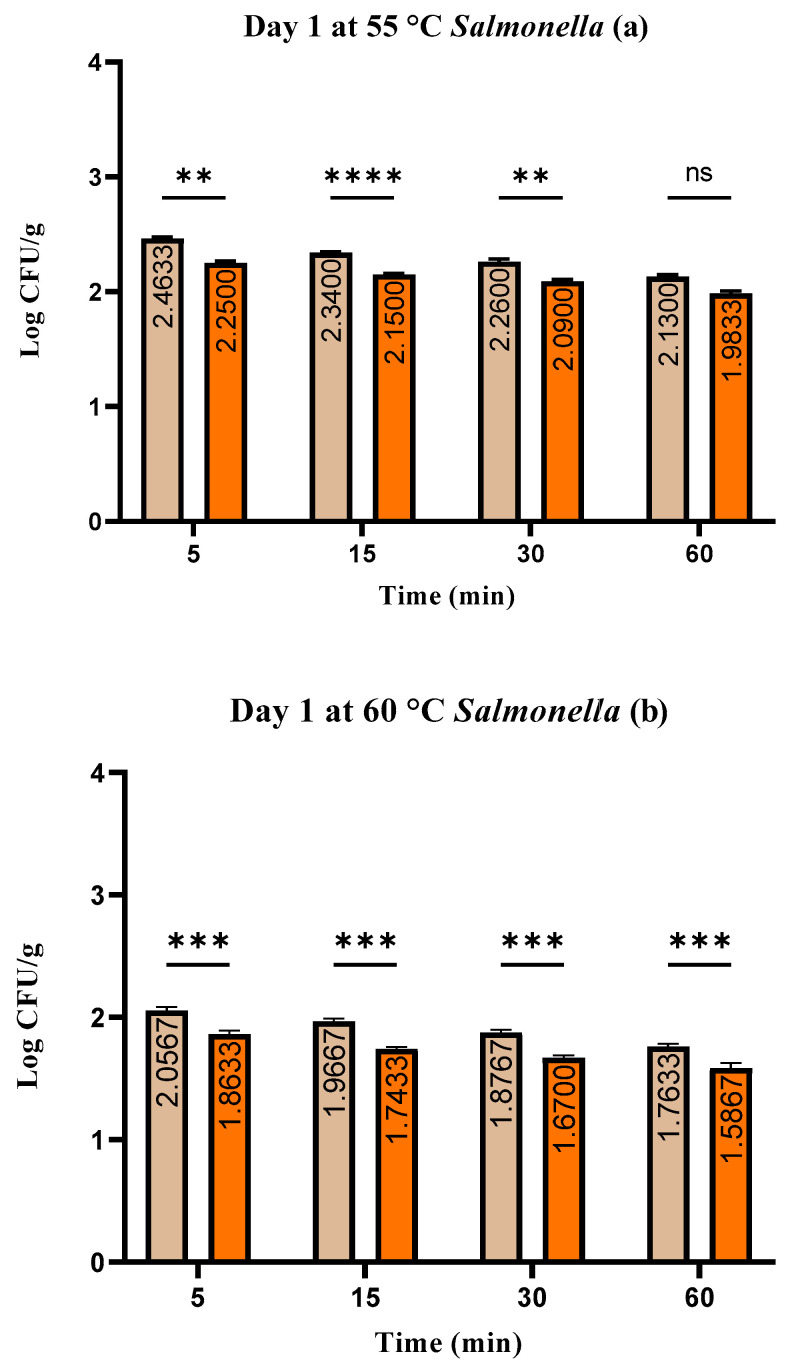

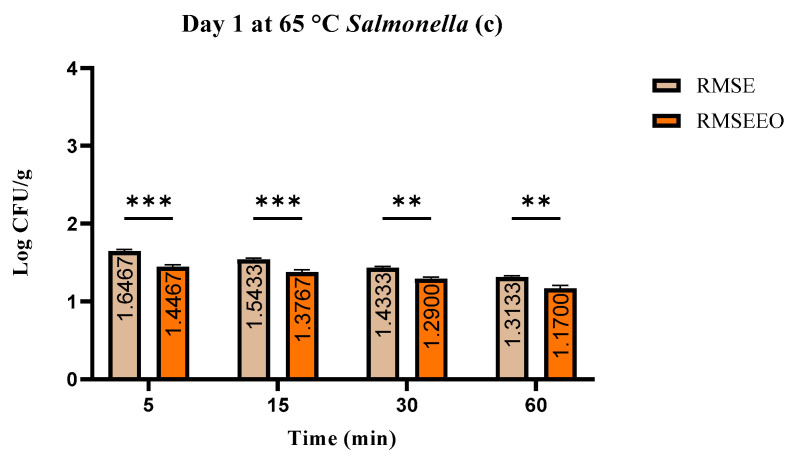
(**a**–**c**). The number of *Salmonella* spp. (log CFU/g) at day 1: Test used: Šídák’s multiple comparisons test; Group (RMSE—RMSEEO), **** *p* ≤ 0.0001, *** *p* ≤ 0.001, ** *p* ≤ 0.01, ns—not significant.

The range of *S. enterica* log CFU/g in the group inoculated with *S. enterica* was 1.31 ± 0.03 to 2.46 ± 0.05, in the wild thyme EO-treated group it was 1.17 ± 0.06 to 2.25 ± 0.05. The log CFU/g of *Salmonella* varied from 1.17 ± 0.06 to 2.46 ± 0.05 (Figure 3a–c).

**Figure 4:**
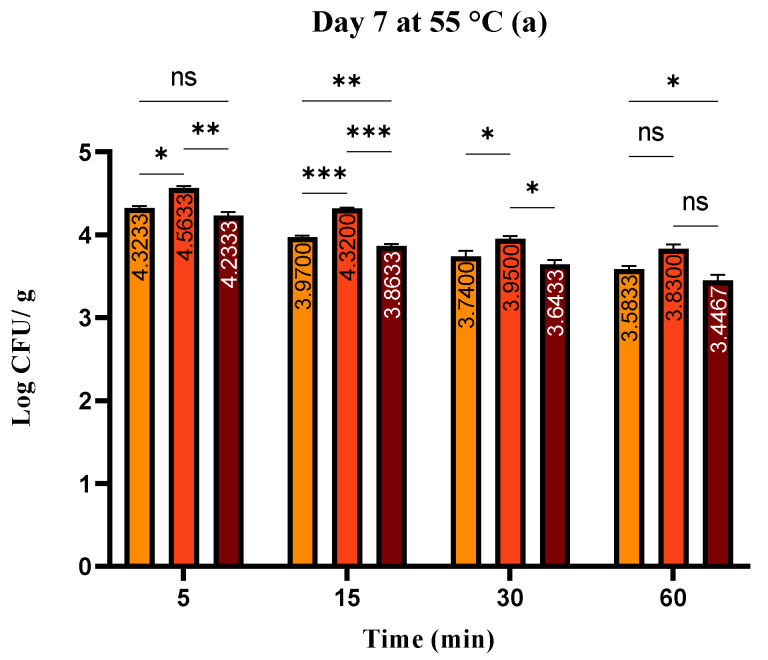

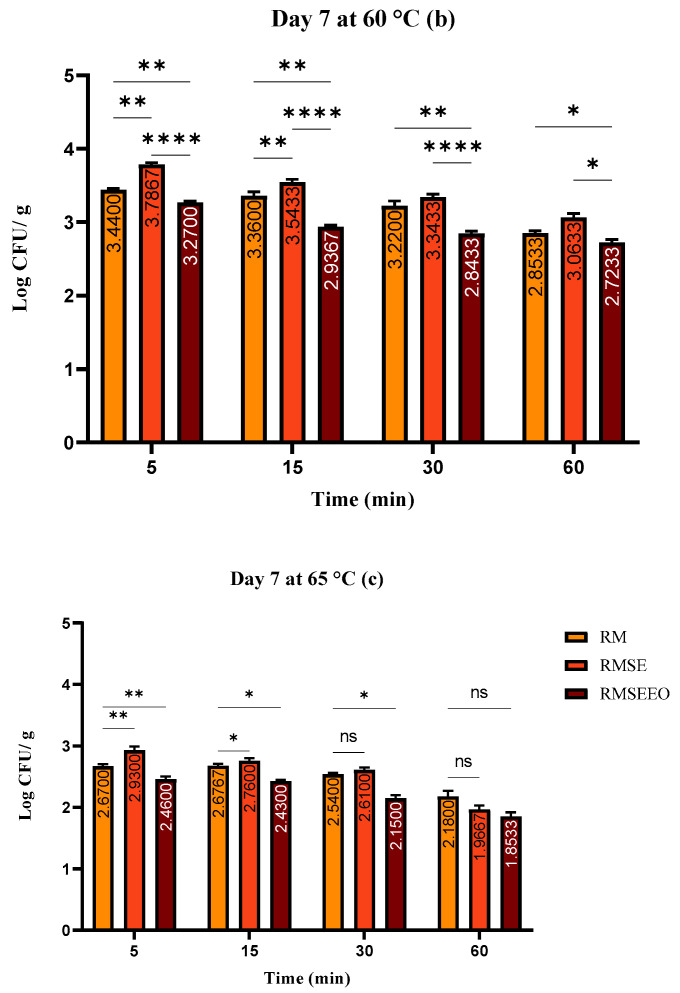
(**a**–**c**). Total count of bacteria (log CFU/g) at day 7: Tukey’s multiple comparisons test, Groups (RM vs. RMSE, RM vs. RMSEEO, and RMSE vs. RMSEEO). **** *p* ≤ 0.0001, *** *p* ≤ 0.001, ** *p* ≤ 0.01, * *p* ≤ 0.05, ns—not significant.

Between 1.97 ± 0.07 and 4.56 ± 0.02 log CFU/g in the *S. enterica* treatment groups, between 1.85 ± 0.02 and 4.23 ± 0.03 in the *S. enterica* treatment groups and *T. serpyllum* EO, and between 2.85 ± 0.06 and 4.32 ± 0.03 log CFU/g in the control groups was the range of total bacterial counts on the 7th study day (Figure 4a–c).

**Figure 5:**
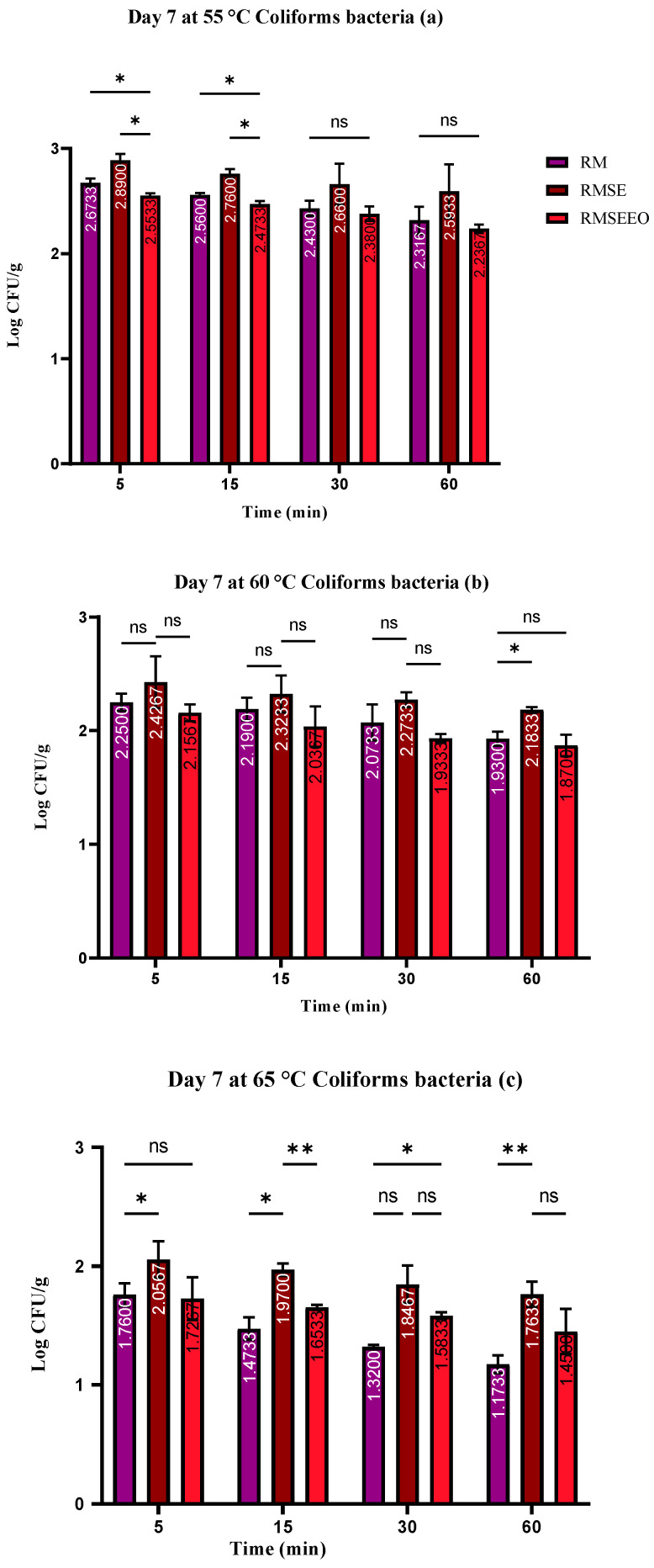
(**a**–**c**). Number of coliform bacteria (log CFU/g) at day 7: Tukey’s multiple comparisons test; (RM vs. RMSE, RM vs. RMSEEO, and RMSE vs. RMSEEO). ** *p* ≤ 0.01, * *p* ≤ 0.05, ns—not significant.

**Figure 6:**
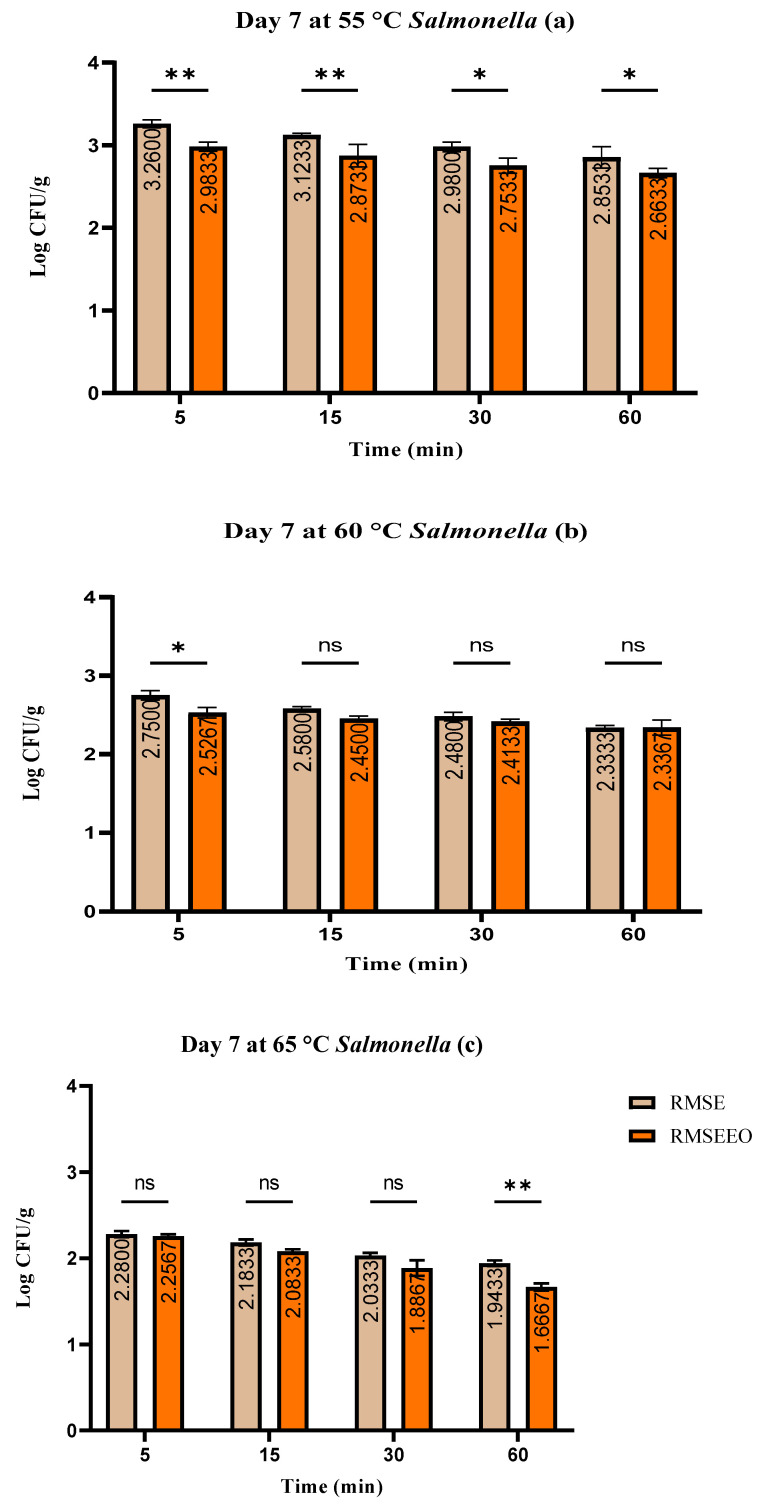
(**a**–**c**). The number of *Salmonella* spp. (log CFU/g) at day 7: Test used: Šídák’s multiple comparisons test; Group (RMSE—RMSEEO), ** *p* ≤ 0.01, * *p* ≤ 0.05, ns—not significant.

The number of coliform bacteria varied in the *S. enterica* treatment groups between 1.76 ± 0.03 log CFU/g and 2.89 ± 0.04 log CFU/g, in the *S. enterica* treatment group and *T. serpyllum* EO, it was between 1.45 ± 0.06 log CFU/g and 2.53 ± 0.05 log CFU/g, and in control groups, it was between 1.17 ± 0.03 log CFU/g and 2.67 ± 0.05 log CFU/g (Figure 5). Within the treated groups, the amount of *S. enterica* varied between 1.67 ± 0.07 and 3.26 ± 0.05 log CFU/g (Figure 6).

### 3.2. Identified Species of Bacteria at Days 1 and 7

**Figure 7:**
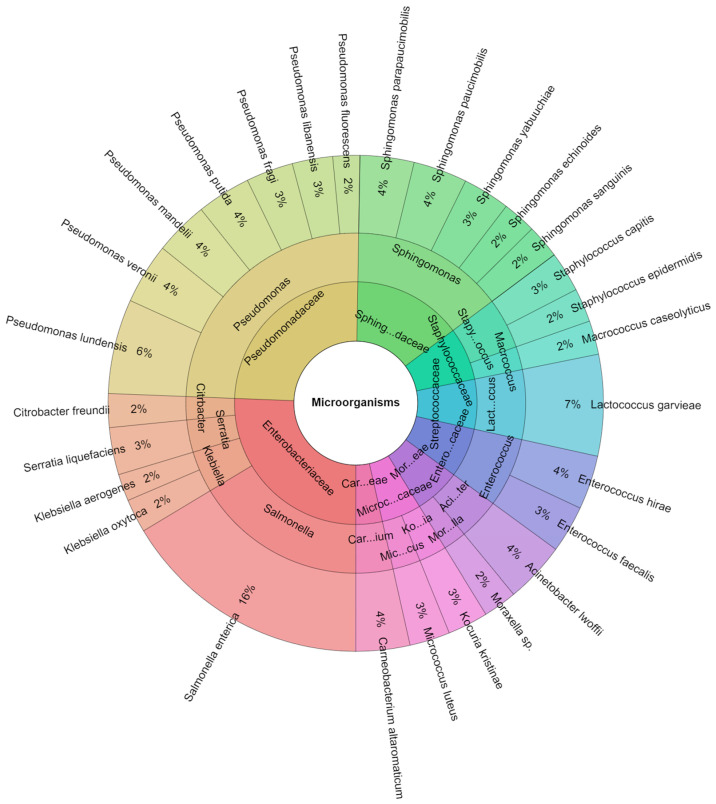
Krona chart: percentage structure of isolated species of microorganisms from rabbit samples at day 1.

A total of 227 species were isolated from rabbit meat samples in all groups in one day. From all isolates, 10 families, 15 genera, and 29 species were identified. The most isolated species were *Salmonella enterica* (16%), the strain was inoculated on the rabbit meat samples, and *Lactococcus garvieae* (7%) from all groups (Figure 7).

**Figure 8:**
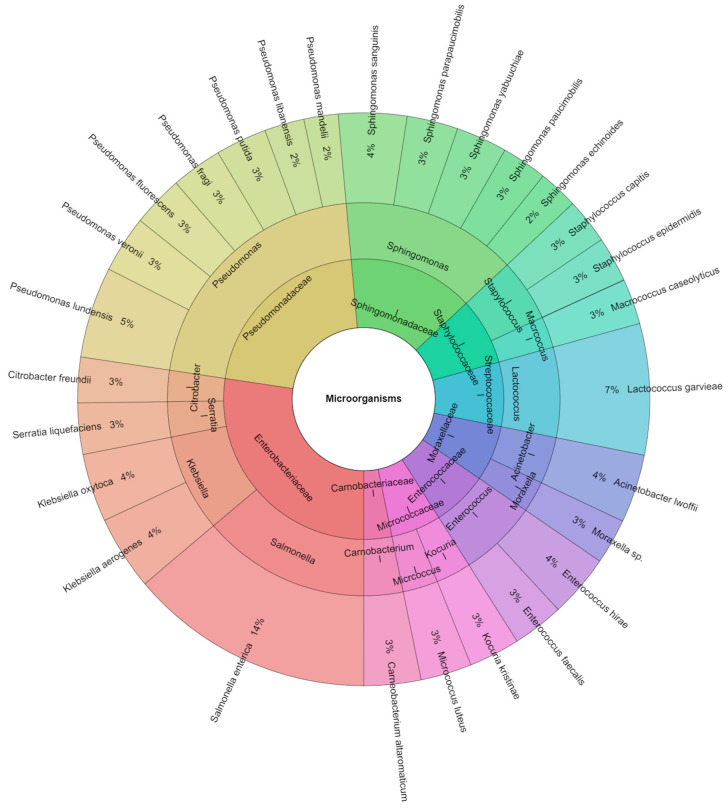
Krona chart: percentage structure of isolated species of microorganisms from rabbit samples over 7 days.

A total of 311 species were isolated from rabbit meat samples in all groups over seven days. From all isolates, 10 families, 15 genera, and 29 species were identified. The most isolated species were *Salmonella enterica* (14%) and *Lactococcus garvieae* (7%) from all groups (Figure 8).

## 4. Discussion

In our investigation, we subjected rabbit meat to *S. enterica* and *Tymus serpyllum* EO, employing varying temperatures and durations. The microbiological quality was found to be satisfactory on the initial day of preparation and remained so after a week of refrigeration. This study specifically delved into the microbial changes in rabbit meat prepared through the sous vide method, chilled at 4 °C for seven days. The outcomes of this research have the potential to enhance the microbiological quality of rabbit meat cooked by sous vide, providing valuable technical insights for the food industry, particularly the ready-to-eat sector. Further research is necessary to establish protocols ensuring the microbiological safety of sous vide-cooked rabbit meat, aiding the food business in enhancing consumer satisfaction. While chemical preservatives have traditionally been effective against harmful germs and food spoilage, the current trend favors healthier options without artificial additives. Hence, the food industry is exploring natural antibacterial compounds like EOs. EOs, derived from plant materials like leaves, seeds, flowers, roots, and twigs, are aromatic liquids. The incorporation of EOs in meat has the potential to inhibit the growth and survival of *S. enterica*, aligning with consumer preferences for natural preservatives [25]. Rabbit meat is considered as a safe product because it has not been linked to any foodborne disease epidemics [27]. On the first day of evaluation, 3.17 ± 0.01 log CFU/g of total count and zero log CFU/g of coliform bacteria were identified in the raw, uncooked rabbit meat. The meat from upscale stores had total bacterial counts that were within the permissible range (4.8 log CFU/cm^2^), suggesting that the meat customers bought there was safe to eat [28]. Nevertheless, rabbits can harbor toxic substances from various origins, including skin, intestinal contents, excretions, the slaughterhouse setting, personnel, as well as the practices related to butchering and retail packaging and handling, akin to other raw meats. Similar to the handling of carcasses from other meat animals, rabbit carcasses undergo collection, processing, and preservation. The post-mortem biochemical alterations in rabbit muscle closely resemble those observed in red meat and poultry [29]. Consequently, the spoilage primarily induced by microorganisms is fundamentally akin to that observed in refrigerated meat. Forty New Zealand white rabbits, comprising twenty freshly slaughtered rabbits from an experimental farm and twenty processed rabbit carcasses from supermarkets, underwent bacterial testing. The average aerobic plate counts for recently slaughtered rabbits at temperatures of 37 °C and 1 °C, along with the counts for Enterobacteriaceae, *Pseudomonas*, and *Staphylococcus*, were 10^4^ ± 2 × 10^3^, 8 × 10^2^ ± 10^2^, 6 × 10^2^ ± 10^2^, 3 × 10^2^ ± 10^2^, and 10^2^ ± 60 CFU/g, respectively. In comparison, the corresponding figures for processed rabbit carcasses from grocery stores were 8 × 10^5^ ± 3 × 10^4^, 2 × 10^5^ ± 10^4^, 4 × 10^4^± 8 × 10^3^, 2 × 10^4^ ± 6 × 10^3^, and 4 × 10^3^ ± 4 × 10^2^ CFU/g, respectively [30]. In our research, we examined the microbiological characteristics of meat subjected to vacuum packaging and temperature treatment. The findings indicated a reduction in bacterial counts with the use of thyme EO. This outcome may be linked to the chemical composition of the EO, particularly its concentration of phenolic compounds, such as thymol. The results of this investigation provide initial insights that could be valuable for future consumer-oriented studies on rabbit meat prepared through the sous vide cooking technique. Consistently, in sous vide-treated meat, the overall bacterial count across all groups was significantly lower with increased temperature and duration, aligning with findings observed in our study. Sous vide treated samples (63–65 °C, 60 min—2.5 log CFU/g) had a lower TVC than cooked beef (2.8 log CFU/g), according to Soletska and Krasota [31]. After six days of chilling, the TVC of the sous vide samples increased slightly (2.6 log CFU/g), but the TVC of the conventionally cooked samples increased somewhat (3.1 log CFU/g). In Hong et al.’s [32] investigation, a lower sous vide processing temperature (62 °C, 35 min) produced a higher TVC of 4.4 log (CFU/g) on the preparation day; after seven days of storage, the count dropped to 3.5 log CFU/g. Comparable to what we discovered, Bıyıklı et al. [33] discovered that cooking meat at various temperature–time combinations decreased TVC by 2. Our study, utilizing a combination of thyme essential oil (EO) during the sous vide process, indicates an enhancement in the microbiological quality of meat compared to its raw counterpart. This observation aligns with the findings of Wang et al. [27] who demonstrated that vacuum-packed samples exhibited superior microbiological quality in comparison to traditionally cooked samples. Additionally, the elevated temperature during the vacuum treatment led to a reduction in the quantity of microorganisms. It is worth noting that the initial microbial load of the meat in our investigation was substantial. In contrast, commercially available meat, typically transported without a cold chain by consumers and stored at a retail chain for several days, may necessitate more rigorous heat treatment. It is usually not enough to destroy dangerous bacteria, as demonstrated by the study by Bıyıklı et al. [33], where a *Listeria* spp. has been found in cooked meat. In our study rabbit meat was inoculated with the pathogenic bacteria *S. enterica*. Coliform counts were found in all groups following seven days of storage at 4 °C. According to Hong et al. [32], overall coliform levels were generally regarded as satisfactory when they were less than log 2 CFU/g. In our study a lover number less than log 2 CFU/g was found in the group treated with essential oil in sous vide rabbit meat samples at a temperature of 60 °C for 30 min. In other groups, the lowest log 2 CFU/g numbers were found in control samples at a temperature of 60 °C for 60 min. In the *Salmonella*-treated group, the count of coliform bacteria exhibited a decline with rising temperature and duration, reaching below log 2 even at 65 °C for 15 min. Furthermore, the investigation revealed that the microbiological condition of vacuum-packed animal products did not indicate the presence of foodborne pathogens such as *L. monocytogenes*, *Clostridium perfringens*, *Bacillus cereus*, *Salmonella*, and other members of the Enterobacteriaceae family [34]. In our research, we introduced *Salmonella enterica* to samples of rabbit meat. When employing sous vide cooking, particular attention is given to bacteria that do not form spores and can thrive in low-oxygen conditions, specifically facultative anaerobic microorganisms like *Salmonella* spp., pathogenic strains of *Escherichia coli*, *Staphylococcus aureus*, *Yersinia enterocolitica*, and *Listeria monocytogenes*. Consequently, the designated minimum acceptable temperature for sous vide cooking is 55 °C for all meat types, excluding poultry, for which the recommended temperature is 60 °C. Regulatory standards in numerous countries specify a combination of temperature and time aimed at achieving a 6.5-log reduction in bacterial load for most pasteurized foods, with the exception of poultry, where a 7-log reduction in *Salmonella* is mandated [35].

In our study, *Salmonella enterica* was present in rabbit meat samples in which the rabbit meat was contaminated at all temperatures and times. Significantly better results were achieved in experimental groups where essential oil was added with *Salmonella enterica*. Currently utilized physical interventions to minimize *Salmonella* on red meat, notably beef, include heating to a temperature between 48 and 66 °C; nonetheless, depending on the serotype, these bacteria have been reported to survive [36,37,38]. In other research, it was found that in high-moisture foods like meat and a model meat juice system, *Salmonella* can survive exposure to heating at temperatures as high as 70 °C [39]. Numerous investigations have been carried out to ascertain *Salmonella*’s heat tolerance on beef and chicken meat at either ≤70 °C or ≥70 °C [38,40,41]. Meat has been the subject of studies employing temperatures ≥ 70 °C for as little as 15 min [40,41]. *Salmonella*’s ability to survive is impacted by the length of heat exposure at 70 °C (47). It has long been believed that thermal inactivation of *Salmonella* in meals, like the foods described in this work, follows first-order kinetics, meaning that the proportion of survivors decreases logarithmically with time [42]. While the interior of intact beef pieces is generally thought to be sterile, there are situations in which *Salmonella* can infiltrate these cuts’ interiors. *Salmonella* in whole muscle was found to be more heat resistant than *Salmonella* in ground meat by Orta-Ramirez et al. [43].

In the past few years, mass spectrometry, particularly MALDI-TOF MS Biotyper, has proven valuable for the precise and sensitive identification of microorganisms. This application extends not only to clinical samples but also encompasses microorganisms derived from non-clinical sources such as plants and agricultural samples. The MALDI-TOF MS Biotyper’s popularity has surged due to its numerous advantages, including swift and accurate identification, cost-effectiveness, and straightforward operation. As a result, it is widely employed for the identification of bacteria and microscopic filamentous fungi [44]. On both days 10 families, 15 genera, and 29 species were identified with MALDI-TOF MS Biotyper from sous vide rabbit meat. The most isolated species were *Salmonella enterica*, *Lactococcus garvieae*, *Pseudomonas lundensis*, *Acinetobacter lwoffii*, *Pseudomonas veronii*, *Sphingomonas sanguinis*, *Klebsiella aerogenes*, and *Klebsiella oxytoca*. In Canada, an experimental investigation was carried out to assess the influence of incorporating a commercial product containing EO (XtractTM) and plant extracts (onion and cranberry) into the rabbit diet on meat quality. Each of the five groups, consisting of 48 weaned female Grimaud rabbits, received either a standard diet or a diet supplemented with 500 or 1000 ppm of onion extract, 500 ppm of cranberry extract, and 100 ppm of essential oil either individually or in combination. The microbiological quality of whole hind paws stored under both aerobic and anaerobic conditions at 4 °C was analyzed. Growth performance, feed intake, composition, and meat quality were similar across all experimental groups. However, the groups receiving dietary supplements exhibited significantly higher total meat phenolic content. The influence of these supplements was evident, with a notable enhancement in microbial control observed under anaerobic conditions, especially for total aerobic mesophile counts, putative *Pseudomonas* spp., and Enterobacteriaceae. Overall, the most effective inhibition of microbial growth was achieved through the treatment with onion extract (500 ppm) [45]. Freshly slaughtered rabbits had a lower bacterial load than processed rabbit corpses from supermarkets. From processed rabbit carcasses, *Salmonella* Typhimurium, *Pseudomonas aeruginosa*, and *Staphylococcus aureus* were recovered together with *Escherichia coli* and *Listeria monocytogenes*. None of the analyzed samples included a solitary case of *Yersinia enterocolitica* [30]. The different tested additives had no effect on the growth of Enterobacteriaceae, *Pseudomonas* spp., lactic acid bacteria, or other aerobic psychotropic bacteria in rabbit meat burgers. In the same study, wild garlic and garlic powder were added to rabbit meat burgers to extend their shelf life and improve their eating quality [46]. In our research aimed at prolonging the shelf life of sous vide rabbit meat, we employed *Thymus serpyllum* to combat *S. enterica*. Our findings revealed the evident antimicrobial activity of wild thyme essential oil against *S. enterica* bacteria, particularly as temperatures and exposure time increased. Previous research [19,47,48,49] evaluated the use of thyme plant extracts in meat and dairy products. According to El Abed et al. [47], the addition of *Thymus capitata* EO to minced beef samples at doses of 0.25 and 1.0% effectively inhibited the growth of bacteria. Furthermore, Bošković et al. [19] found that minced pork vacuum-packed or MAP and held at 3 °C for 15 days effectively inhibited the growth of *Salmonella* spp. at a concentration of 0.6% in thyme EO. *Thymus capitatus* and *Thymus algeriensis* EOs (3%) were added to minced beef in a study by Jayari et al. [49], and antibacterial activity has been demonstrated against pathogenic bacteria (*Escherichia coli*, *Salmonella Typhimurium*, *Staphylococcus aureus*, and *Pseudomonas aeruginosa*). Furthermore, Pesavento et al. [50] validated the antibacterial effects of volatiles in meat products by incorporating EO into meatballs. Their findings indicated that concentrations of 1% and 2% of thyme EO progressively diminished the microbial load in the samples, while the 0.5% concentration of thyme EO exhibited a bacteriostatic effect against *Listeria monocytogenes* at 4 °C. According to Barbosa et al. [51] at 5 °C, thyme EO added to minced beef that had been exposed to radiation had a bacteriostatic impact on *Salmonella enteritiditis*, *Listeria monocytogenes*, *Staphylococcus aureus*, and *Escherichia coli*. Amariei et al. [52] found that thyme, an EO (0.5–1.5%), had a comparable impact on the minced meat’s microbiological stability. Prior research has demonstrated that thyme EO has negligible or no antibacterial activity when studied in vivo. For example, Solomakos et al. [53] found that the addition of 0.6% (*v*/*w*) EO to minced beef meat at 4 °C did not inhibit the growth of *Escherichia coli* O157:H7 strains. Nevertheless, the populations of the tested pathogens were markedly reduced (approximately 4.8 log CFU/g) at 10 °C compared to the control group (approximately 7.1 log CFU/g), suggesting that the antibacterial efficacy of this EO was influenced by temperature. Similarly, Gouveia et al. [54] found that the presence of thyme EO in sous vide cook–chill beef samples at the minimum inhibitory concentration (MIC level) of 0.39 *v*/*v* did not significantly alter the density of *Listeria monocytogenes* at 2 °C or 8 °C, as compared to the samples without EO. In such situations, higher EO concentrations might be necessary in real food matrices to achieve the same level of inactivation observed in vitro. However, such elevated concentrations often lead to undesirable changes in color and flavor in food products, affecting their sensory characteristics [55,56].

## 5. Conclusions

A growing source of premium animal protein with significant nutritional value for additional nutrients is rabbit meat. Nevertheless, rabbit meat is also thought to be a possible source of food poisoning microorganisms, which can reduce the meat’s shelf life and cause a number of harmful health effects. Interestingly, several studies have shown that adding natural additives like wild thyme EO or using physical techniques such as vacuum packing can enhance the microbiological quality of rabbit meat. By using the sous vide process, meat can have a longer shelf life and higher nutritional content. Nevertheless, the remaining temperature–time combinations effectively inactivated the bacteria, including *Salmonella enterica*. The findings of our investigation were also useful in the identification of microorganisms in rabbit meat that was cooked, chilled, and prepared by the sous vide method. Mass spectrometric analysis of rabbit meat revealed a diverse microbiota. The presence of *Acinetobacter lwoffii*, *Lactococcus garvieae*, *Pseudomonas lundensis*, *Sphingomonas sanguinis*, and other well-known species was detected, in addition to *S. enterica*, which was applied to the meat. Rabbit meat is safer for storage if cooked by the sous vide method and chilled with thyme EO. Subsequently, we found that *S. enterica* was positively affected by thyme EO at a concentration of 1% (*v*/*w*). Temperature had an effect on the behavior of *S. enterica*; therefore, after 7 days of storage at 4 °C, the effect of rabbit meat samples containing thyme EO grew. In addition, the antibacterial activity of thyme EO used in sous vide rabbit meat may vary depending on the amount used. The findings of this study may be useful for food manufacturers as they suggest that thyme EO can be used as a natural preservative to prevent the growth of *S. enterica* in sous vide chook–chill rabbit meat and increase their safety during storage at appropriate refrigeration temperatures.

## Data Availability

All data generated or analyzed during this study are included in this published article.
